# Respiration and substrate transport rates as well as reactive oxygen species production distinguish mitochondria from brain and liver

**DOI:** 10.1186/s12858-015-0051-8

**Published:** 2015-09-10

**Authors:** Aaron M. Gusdon, Gabriel A. Fernandez-Bueno, Stephanie Wohlgemuth, Jenelle Fernandez, Jing Chen, Clayton E. Mathews

**Affiliations:** Department of Pathology, Immunology, and Laboratory Medicine, The University of Florida College of Medicine, Gainesville, FL 32610 USA; Department of Neurology, Weill Cornell Medical Center/NewYork-Presbyterian Hospital, New York, NY 10065 USA; Department of Animal Sciences, Institute of Food and Agricultural Sciences, The University of Florida, Gainesville, FL 32610 USA; Present address: 1275 Center Dr, Room J597, P.O. Box 100275, Gainesville, FL 32610-0275 USA

## Abstract

**Background:**

Aberrant mitochondrial function, including excessive reactive oxygen species (ROS) production, has been implicated in the pathogenesis of human diseases. The use of mitochondrial inhibitors to ascertain the sites in the electron transport chain (ETC) resulting in altered ROS production can be an important tool. However, the response of mouse mitochondria to ETC inhibitors has not been thoroughly assessed. Here we set out to characterize the differences in phenotypic response to ETC inhibitors between the more energetically demanding brain mitochondria and less energetically demanding liver mitochondria in commonly utilized C57BL/6J mice.

**Results:**

We show that in contrast to brain mitochondria, inhibiting distally within complex I or within complex III does not increase liver mitochondrial ROS production supported by complex I substrates, and liver mitochondrial ROS production supported by complex II substrates occurred primarily independent of membrane potential. Complex I, II, and III enzymatic activities and membrane potential were equivalent between liver and brain and responded to ETC. inhibitors similarly. Brain mitochondria exhibited an approximately two-fold increase in complex I and II supported respiration compared with liver mitochondria while exhibiting similar responses to inhibitors. Elevated NADH transport and heightened complex II–III coupled activity accounted for increased complex I and II supported respiration, respectively in brain mitochondria.

**Conclusions:**

We conclude that important mechanistic differences exist between mouse liver and brain mitochondria and that mouse mitochondria exhibit phenotypic differences compared with mitochondria from other species.

**Electronic supplementary material:**

The online version of this article (doi:10.1186/s12858-015-0051-8) contains supplementary material, which is available to authorized users.

## Background

Mitochondrial dysfunction has been implicated in a growing number of disorders. The etiologies of these syndromes have been associated with an imbalance in mitochondrial reactive oxygen species (ROS) production, which consists principally of the generation of superoxide and hydrogen peroxide. Mitochondrial ROS production has been well characterized in neurodegenerative conditions, including Alzheimer’s disease [1–3], Parkinson’s disease [1, 3–5], amyotrophic lateral sclerosis [1, 6], and Huntington’s disease [1, 2, 7]. The dysfunction of β cells in both type 2 and type 1 diabetes [8–11], has been linked to mitochondrial ROS production and increased superoxide production has been shown to cause DNA damage leading to poly(ADP-ribose) polymerase activation, subsequently Glyceraldehyde 3-phosphate dehydrogenase inhibition, as well as induction of the main pathways of hyperglycemia induced pathology [12]. ROS generated by the mitochondria have also been implicated in the aging process [13–16] as well as in cardiovascular disorders such as hypertension [17–19], atherosclerosis [20–25], and myocardial infarction [26, 27].

Many studies have sought to determine the mechanisms of mitochondrial ROS production. Inhibitors that act on different sites of the electron transport chain (ETC) have been extensively used to localize and quantify mitochondrial ROS production. Complex I and III redox centers have been implicated as the major sites of mitochondrial ROS production [28–30], with recent data suggesting complex II is also capable of producing ROS [31, 32]. Within complex I, both the flavin mononucleotides (FMN) and a distal site, presumably the ubiquinone binding site, have been shown to be capable of generating ROS with the direction of electron flow dictating the relative contribution from each site [33]. The location of complex III supported ROS production has been shown to be primarily the cytochrome bc_1_ complex promoted by a partially oxidized ubiquinone pool [34]. However, the FMN site within complex I has been shown to be responsible for the majority of ROS production under ATP generating conditions [35].

There is reason to believe that mitochondria from different mouse tissues exhibit unique functional characteristics. Studies using isolated rat mitochondria have observed variation in the activity of the ETC complexes comparing tissues [36]. The MitoCarta database has revealed that in mouse tissues many nuclear encoded mitochondrial proteins have unique tissue specific expression [37]. Further, it has been shown that liver mitochondria require less Ca^2+^ than brain mitochondria to initiate the mitochondrial permeability transition and mouse brain mitochondria were found to have a more robust ROS increase in response to complex III inhibitors than rat brain mitochondria [38]. These differences are not unexpected given the growing knowledge of the importance of signaling between the nuclear and mitochondrial genomes. Nuclear genes are potentially targeted for expression either by changes in the release of signaling molecules from the mitochondria (retrograde signaling) or by communication of nuclear gene products with proteins encoded by mitochondrial genes (intergenomic interactions) [9, 10, 39]. Indeed, mitochondrial DNA (mtDNA) haplogroups impact ROS production [9, 10] activating compensatory mechanisms resulting in the normalization of mitochondrial respiration [40]. While mice represent some of the most widely used models of disease, there is a lack of information comparing the parameters of mitochondrial function from different mouse tissues.

We hypothesize that mouse mitochondria will exhibit tissue specific phenotypes. In this report, the effects of some of the most commonly used ETC. inhibitors-rotenone, *p*-chloromercuribenzoate (CMB), diphenyleneiodonium (DPI), antimycin A, and myxothiazol-were tested on various functional parameters of isolated mouse brain and liver mitochondria. These two tissues exhibited some similarities in mitochondrial function, but there were notable differences in respiration and ROS production. The effect of the panel of inhibitors demonstrated further divergence between the mitochondria isolated from these two tissues.

## Results

### Method of isolation does not alter function or purity of liver mitochondria

**Fig. 1:**
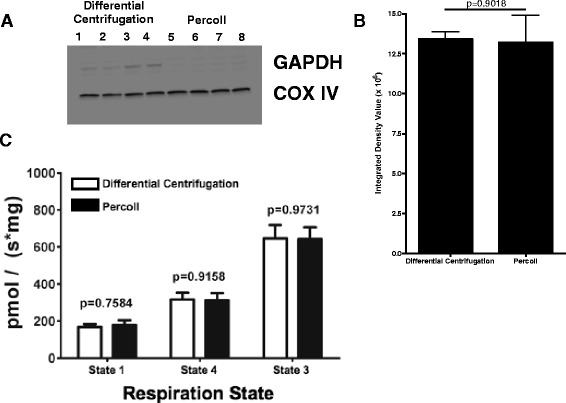
Purity and function of liver mitochondria comparing isolation with differential centrifugation versus a Percoll gradient. **a** Western blot. Lanes 1–4 are liver mitochondria isolated by differential centrifugation. Lanes 5–8 are liver mitochondria isolated using a Percoll gradient. Protein was loaded at 25 μg/lane. COX IV antibody [20E8] (Abcam) was used to detect mitochondrial protein. GAPDH, an enzyme expressed in the cytosol, demonstrates minimal differences in cytosolic contamination among the preparations. **b** Densitometry analysis of the immunoblots shows there is no difference in the amount of COX IV protein in the mitochondrial preparations resulting from these two methodologies. **c** Respiration. Liver mitochondria were isolated by either differential centrifugation (open bars) or with Percoll (closed bars) and then tested for respiratory capacity. Mitochondrial respiration was measured using the complex I substrates glutamate and malate. State 1 respiration was recorded without substrates, subsequently state 2 respiration was recorded after the addition of substrates, and finally ADP (0.5 mM final concentration) was added in order to induce state 3 respiration. *n* = 6 for both mitochondrial preparations and for all three respiratory states. Respiratory rates were comparable for the two mitochondria isolated by the two methods without significant differences

Historically mitochondria have been isolated from liver using differential centrifugation [41] and from brain using a Percoll gradient [42]. To examine if liver mitochondria isolated by these two methods would have measurable differences in functional parameters, we isolated liver mitochondria using either differential centrifugation or Percoll and assayed these preparations for purity and respiration. For these experiments livers were removed, a lateral bisection was performed, and each half was subjected to a different procedure of mitochondria isolation. Liver mitochondria isolated by the two methods had equal purity as assessed by Western blot analysis of COX IV protein abundance (Fig. 1a, b), and did not differ in state two and three respiration (Fig. 1c) when normalized for protein content. As the mitochondria resulting from these two isolation methods were equal in purity and characteristics of mitochondrial function, all other experimental data in this report were derived using liver mitochondria isolated by differential centrifugation for comparison to the historical literature.

### Mitochondrial reactive oxygen species production

ROS production was assessed for intact brain and liver mitochondria in the presence of electron transport chain inhibitors. In order to determine the optimal inhibitor concentration, titration curves were performed for each inhibitor at concentrations of 0, 0.1, 1, 5, 10, and 20 μM in brain and liver mitochondria utilizing either glutamate and malate or succinate to support ROS production (Additional file 1: Table S1). ROS production of brain mitochondria utilizing glutamate and malate was maximal with 5 μM of each inhibitor and without significant differences among 5, 10, and 20 μM of each inhibitor (Additional file 1: Table S1). Addition of inhibitors slightly decreased liver mitochondrial ROS production utilizing glutamate and malate with maximal inhibition reached at 5 μM and without significant difference among 5, 10, and 20 μM of each inhibitor (Additional file 1: Table S1). The decrease in brain mitochondrial ROS production utilizing succinate was maximal by 5 μM of each inhibitor without significant difference among 5, 10, and 20 μM of each inhibitor (Additional file 1: Table S1). A dose response curve of 0–12.8 μM DMSO was performed for brain and liver mitochondrial utilizing either complex I or complex II substrates without significant differences among the concentrations used (Additional file 1: Table S2).

### Reactive oxygen species production by brain mitochondria

**Fig. 2:**
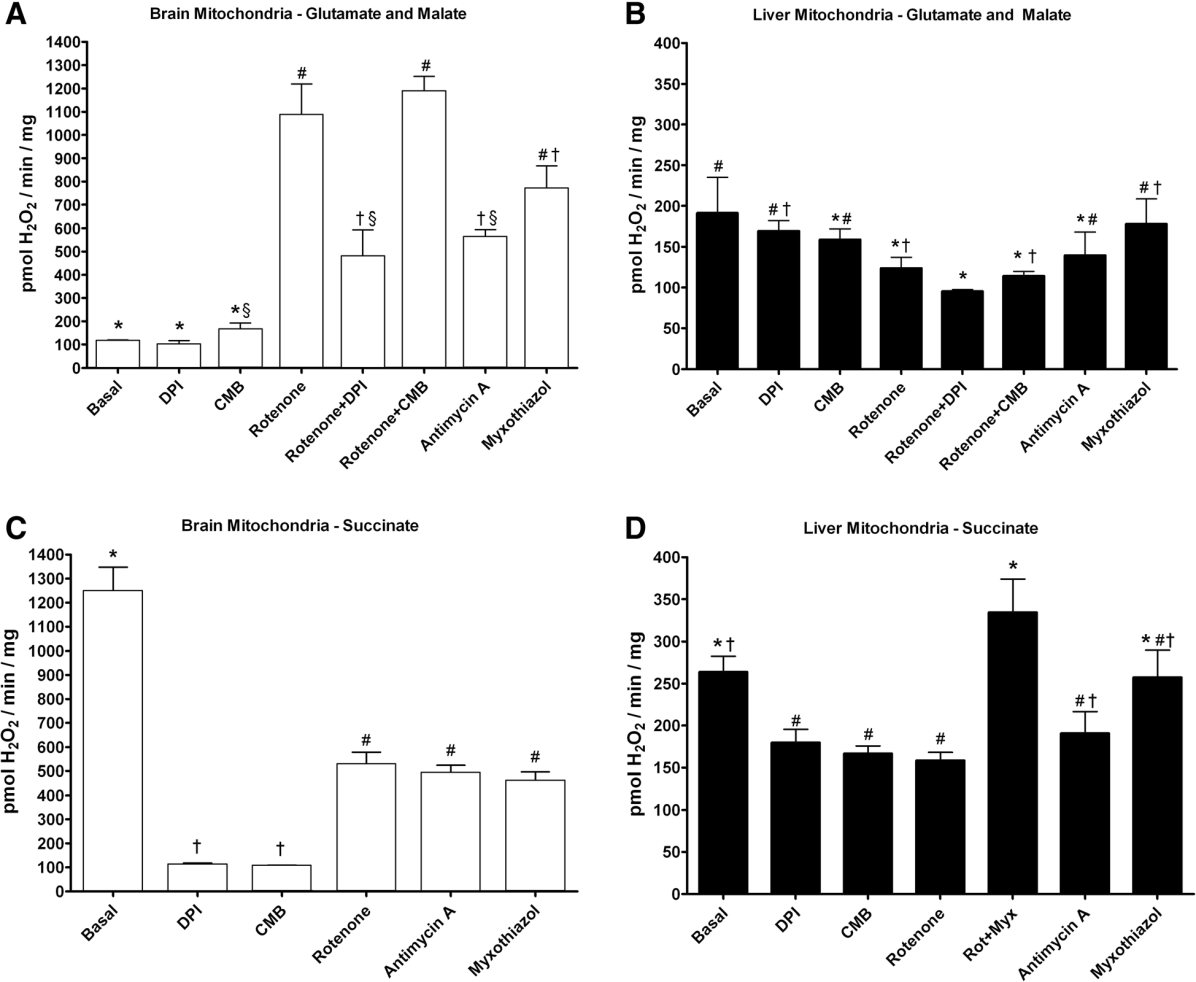
Response of reactive oxygen species production to ETC inhibitors differs between brain and liver mitochondria. Brain (**a**) and liver (**b**) mitochondrial ROS production was assessed utilizing the complex I substrates glutamate and malate. Basal ROS production was recorded for three minutes. After this time, DPI (10 μM), CMB (10 μM), rotenone (10 μM), rotenone + DPI (10 μM each), rotenone + CMB (10 μM each), antimycin A (10 μM), or myxothiazol (10 μM) was added to the reaction and ROS production was recorded for an additional two minutes. Brain (**c**) and liver (**d**) mitochondrial ROS production was assessed utilizing the complex II substrate succinate. Basal respiration was recorded for three minutes. After this time, DPI (10 μM), CMB (10 μM), rotenone (10 μM), antimcyin A (10 μM), myxothiazol (10 μM), or rotenone + myxothiazol (10 μM each; liver only) was added to the reaction, and ROS production was recorded for an additional two minutes. 150 μg of isolated mitochondria was used for each experiment. For each comparison, * vs. § < 0.05; * vs. # < 0.001; * vs. † < 0.01; § vs. # < 0.01, § vs. † < 0.01; # vs. † < 0.05. For each condition, *n* ≥ 3

For brain mitochondria utilizing the complex I substrates glutamate and malate, basal ROS production was unchanged by the addition of DPI, while CMB alone resulted in a slight but statistically significant increase (Fig. 2a). As expected [43], the addition of rotenone increased ROS production from brain mitochondria by 1000 % compared to the basal level. To suppress the rotenone-induced elevation in ROS production, DPI and CMB were added in combination with rotenone. Addition of DPI decreased the rotenone induced increase by half, whereas the inhibitor CMB resulted in a slight but non-significant increase in ROS production. The complex III inhibitors antimycin A and myxothiazol increased ROS production by approximately 450 and 650 % from basal level, respectively (Fig. 2a).

Basal ROS production from brain mitochondria respiring with succinate was characteristically high and 1000 % higher than ROS during the utilization of complex I substrates (1245 ± 288 pmol H_2_O_2_ min^−1^ mg^−1^ [with succinate] versus 114 ± 17 pmol H_2_O_2_ min^−1^ mg^−1^ [with glutamate and malate]; *p* < 0.0001). Addition of the Complex I inhibitors DPI and CMB each significantly decreased succinate-mediated ROS production by greater than 90 %, while rotenone significantly lowered ROS production by roughly 1-half (Fig. 2c). The presence of the complex III inhibitors antimcyin A or myxothiazol decreased this basal ROS signal to a similar extent as rotenone (Fig. 2c).

### Reactive oxygen species production by liver mitochondria

For liver mitochondria utilizing glutamate and malate as substrates, neither DPI nor CBM altered basal ROS production (Fig. 2b). However, rotenone significantly decreased ROS production. The addition of either DPI or CMB to rotenone-inhibited mitochondria had no further significant inhibitory effects compared to rotenone alone. The complex III inhibitors antimycin A and myxothiazol had no significant effect on ROS production (Fig. 2b).

Liver mitochondria utilizing succinate exhibited a slight yet significant increase in basal ROS production compared to liver mitochondria using glutamate and malate (264 ± 37 pmol H_2_O_2_ min^−1^ mg^−1^ [with succinate] versus 181 ± 24 pmol H_2_O_2_ min^−1^ mg^−1^ [with glutamate and malate]; *p* = 0.009). The inhibitors rotenone, DPI, CMB, and antimycin A each resulted in an approximately 15–20 % reduction in ROS production. Myxothiazol had no significant effect. The combination of rotenone and myxothiazol resulted in a slight but non-significant increase in ROS production (Fig. 2d).

### Comparison of brain and liver mitochondrial ROS production

Comparing brain and liver mitochondria utilizing glutamate and malate (Fig. 2 a-basal versus b-basal), liver mitochondria produced more ROS than brain mitochondria (*p* = 0.0002). For both liver and brain mitochondria, DPI had no significant effect on ROS production. CMB resulted in a slight but non-significant increase in brain ROS production yet had no effect on liver ROS production. Rotenone drastically increased ROS production from brain mitochondria yet decreased liver mitochondrial ROS production. The addition of DPI to rotenone inhibited the increase in ROS production by brain mitochondria, and led to a further decrease of liver mitochondrial ROS. The combination of rotenone and CMB had no further effect on ROS production from either brain or liver mitochondria compared with rotenone alone. Considering complex III inhibitors in the context of glutamate and malate supported ROS production, antimycin A increased brain ROS, yet this inhibitor did not affect liver mitochondrial ROS production. Likewise, addition of myxothiazol increased brain ROS production, but did not alter liver mitochondrial ROS production.

Comparing brain and liver mitochondria utilizing succinate (Fig. 2c, d), brain mitochondria produced significantly more ROS (*p* < 0.0001). DPI and CMB each inhibited the majority of brain mitochondrial ROS production, while both inhibited liver mitochondrial ROS production to a lesser extent. Rotenone and antimcyin A both inhibited ROS production to a slightly greater extent in brain compared with liver mitochondria. However, while myxothiazol inhibited brain ROS production to a significant extent, this inhibitor did not significantly block liver ROS production.

### Complex I enzymatic activity

**Fig. 3:**
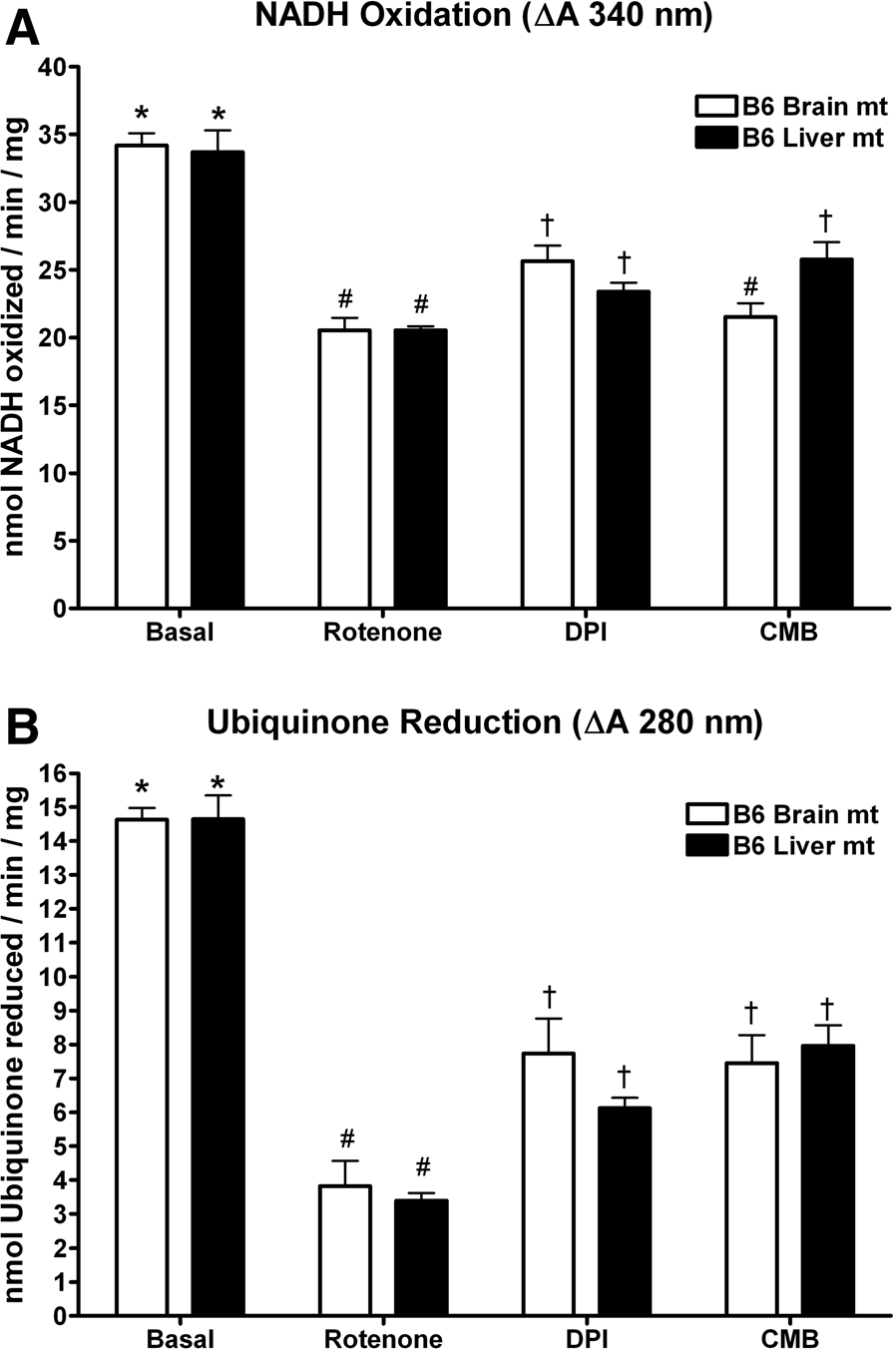
Complex I enzymatic activity is comparable in mitochondrial preparations of brain and liver mitochondria. Mitochondrial complex I enzymatic activity was assessed by following the oxidation of NADH (**a**) or the reduction of ubiquinone (**b**) by 40 μg of isolated brain or liver mitochondria. Complex I activity was measured over three minutes. Basal indicates total complex I activity in the absence of electron transport chain inhibitors. Where indicated, DPI (10 μM), CMB (10 μM), or rotenone (10 μM) was added before the initiation of the reaction. Symbols denote comparisons brain mitochondria treatment groups or liver mitochondria treatment groups, respectively, as well as comparisons between brain and liver mitochondria for each treatment. For each comparison, * vs. # < 0.01; * vs. † < 0.05; # vs. † < 0.05. For each treatment with brain mitochondria *n* = 6. For each treatment with liver mitochondria, *n* = 3

Complex I activity was assessed using both input (by following the oxidation of NADH) and output (by following the reduction of ubiquinone) assays in the presence of complex I inhibitors. Total complex I activity was comparable between brain and liver for both NADH oxidation (Fig. 3a) and ubiquinone reduction (Fig. 3b). DPI inhibited NADH oxidation by approximately 30 % and ubiquinone reduction approximately 50 %, with liver mitochondria being slightly (yet not significantly) more inhibited. CMB inhibited complex I activity to a similar extent as DPI, however brain mitochondria were slightly but significantly (*p* = 0.04) more inhibited than liver mitochondria considering NADH oxidation. Rotenone inhibited the oxidation of NADH by only 40 % (Fig. 3a), whereas it inhibited the reduction of ubiquinone by roughly 75 % with no significant differences between liver and brain (Fig. 3b).

### Complex II and complex III enzymatic activity

**Fig. 4:**
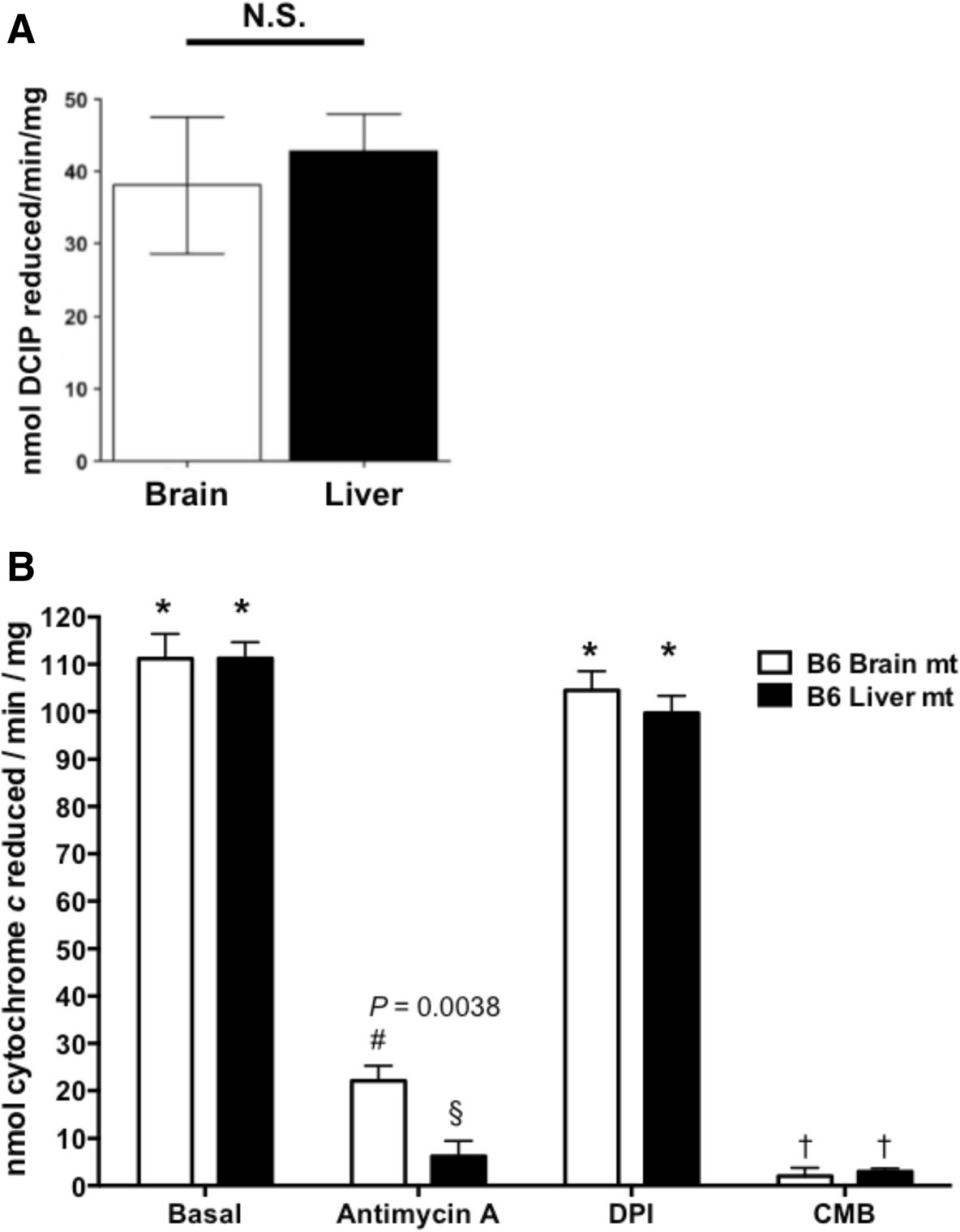
Complex II and III enzymatic activities are similar in mitochondrial preparations of brain and liver mitochondria but differs in response to Antimycin A. **a** Mitochondrial complex II enzymatic activity was assessed by following the reduction of DCIP by 1.5 μg of isolated brain or liver mitochondria over one minute. **b** Mitochondrial complex III enzymatic activity was assessed by following the reduction of cytochrome *c* by 20 μg by isolated brain or liver mitochondria over one minute. Basal indicates the total complex III activity in the absence of electron transport chain inhibitors. When indicated, antimycin A (10 μM), DPI (10 μM), or CMB (10 μM) was added before the initiation of the reaction. Symbols denote comparisons among brain mitochondria treatment groups or liver mitochondria treatment groups as well as comparisons between brain and liver mitochondria for each treatment. For each comparison, * vs. # < 0.01; * vs. † < 0.001; # vs. † < 0.01. For each condition, *n* = 3

Complex II activity in both brain and liver mitochondria was monitored following the reduction of DCIP. The specific activity of complex II was obtained by subtracting the malonate insensitive activity from the total activity. Brain and liver mitochondria showed no significant difference in specific complex II activity (Fig. 4a).

Complex III activity was measured in the presence of complex III and complex I inhibitors. Basal activity was not significantly different comparing liver and brain mitochondria. The complex III inhibitor antimycin A inhibited brain mitochondrial complex III activity by approximately 80 % while liver mitochondria were inhibited by nearly 90 % (*p* = 0.0038). DPI had only a negligible effect on complex III activity for both liver and brain mitochondria. Conversely, CMB nearly completely ablated complex III activity for both liver and brain mitochondria (Fig. 4b).

### Mitochondrial respiration utilizing glutamate and malate

**Fig. 5:**
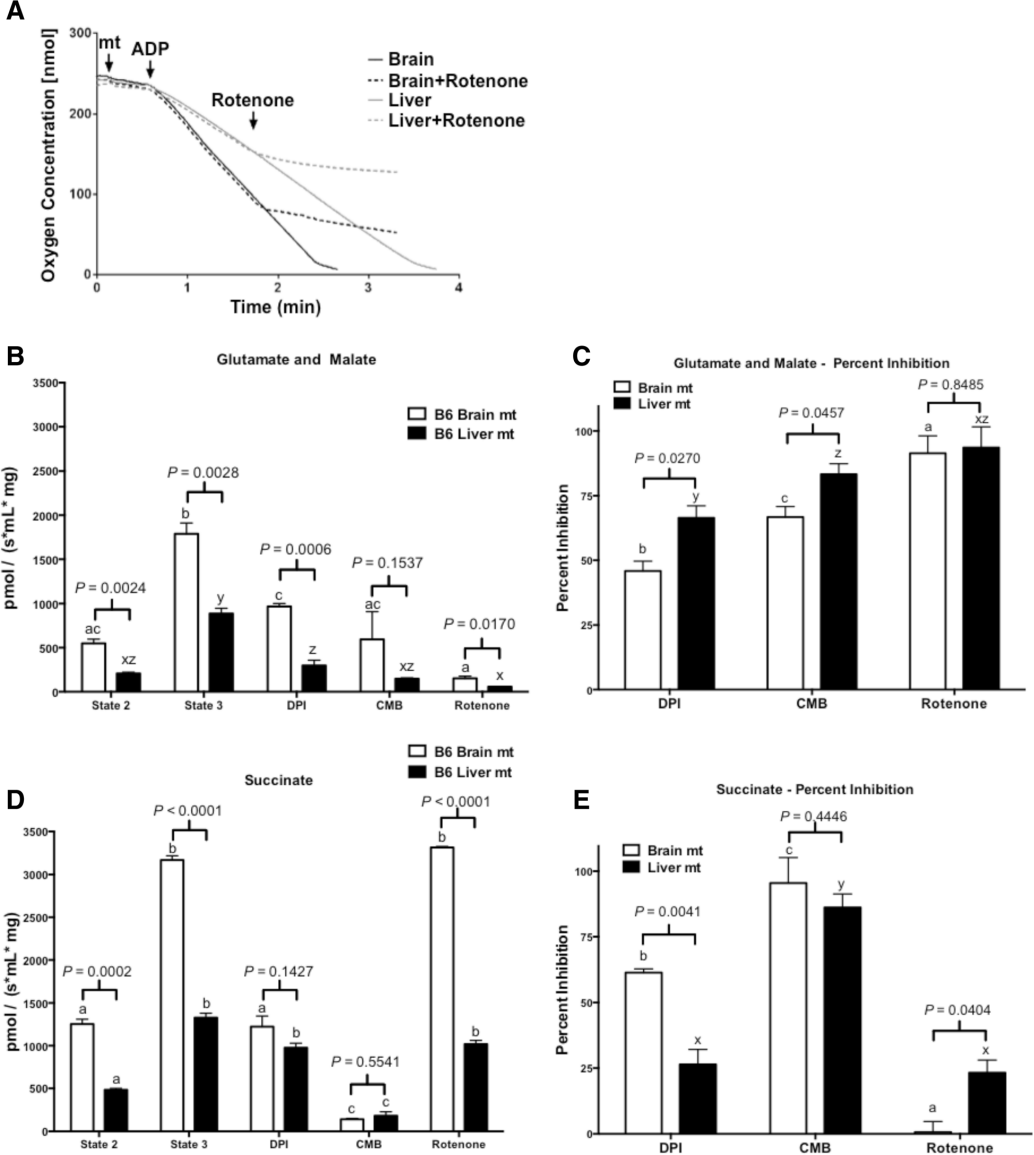
Isolated brain mitochondria exhibit a higher respiratory rate and are differentially affected by ETC. inhibitors compared to liver mitochondria. **a** Representative trace of brain and liver mitochondrial oxygen consumption using glutamate and malate as a substrate with or without addition of rotenone where indicated. (**b**–**d**) Mitochondrial respiration was supported by glutamate and malate (complex I) (**b**, **c**) or succinate (complex II) (**d**, **e**), respectively. State 2 respiration was monitored before the addition of 0.5 mM ADP to induce state 3 respiration. After achieving maximal state 3 respiration, DPI (10 μM), CMB (10 μM), or rotenone (10 μM) was added. Percent inhibition of respiration achieved by rotenone, DPI, and CMB was calculated for both glutamate and malate (**c**) and succinate (**e**) supported respiration by comparing state 3 respiratory rates to stable respiratory rates after inhibitor treatments. For each treatment, n ≥ 3. *P* values are indicated comparing brain and liver mitochondria for state 2 and state 3 respiratory rates, as well as state 3 respiratory rates after rotenone, DPI, or CMB treatment. Isolated mitochondria (0.4 mg) were used for each experiment. For each treatment, brain mitochondria are compared, where a vs. b vs. *c* < 0.05; liver mitochondria are compared, where x vs. y vs. *z* < 0.05

Utilizing glutamate and malate, state 2 and state 3 respiratory rates were more than double for brain compared with liver mitochondria (Fig. 5a, b). Respiratory control ratios (RCR) for isolated brain (4.0 ± 0.3) and liver (4.3 ± 0.68) mitochondria respiring on glutamate and malate were not statistically different (*p* = 0.3). Mitochondrial state 3 respiration was measured in the presence of Complex I inhibitors (Fig. 5b, c). DPI inhibited state 3 respiration by around 50 % in brain and by around 70 % in liver mitochondria. The inhibitory effect of CMB was slightly more pronounced, inhibiting brain mitochondria by nearly 75 % and liver mitochondria by nearly 85 %. Rotenone had a very strong inhibitory effect, lowering state 3 respiration in both brain and liver mitochondria by nearly 95 %.

### Mitochondrial respiration utilizing succinate

Brain mitochondrial state 2 and state 3 respiratory rates were more than double the liver respiratory rates (Fig. 5d). Again, while the isolated brain mitochondria exhibited an elevated rate of respiration there were no differences in the RCR [brain (3.92 ± 0.4), liver (4.1 ± 0.7), *p* = 0.55] suggesting that the mitochondria were equally coupled. Mitochondrial state 3 respiration with succinate was assessed in the presence of Complex I inhibitors (Fig. 5d, e). DPI inhibited brain mitochondrial respiration by roughly 60 %, but inhibited liver mitochondria by only 25 %. CMB had a strong effect, inhibiting brain and liver mitochondrial respiration 96 % and 87 %, respectively. Rotenone had a small effect on brain mitochondria, inhibiting respiration by 12 %; yet liver mitochondrial respiration was significantly inhibited by about 31 %.

### Mitochondrial inner membrane potential (Δψ_m_)

**Fig. 6:**
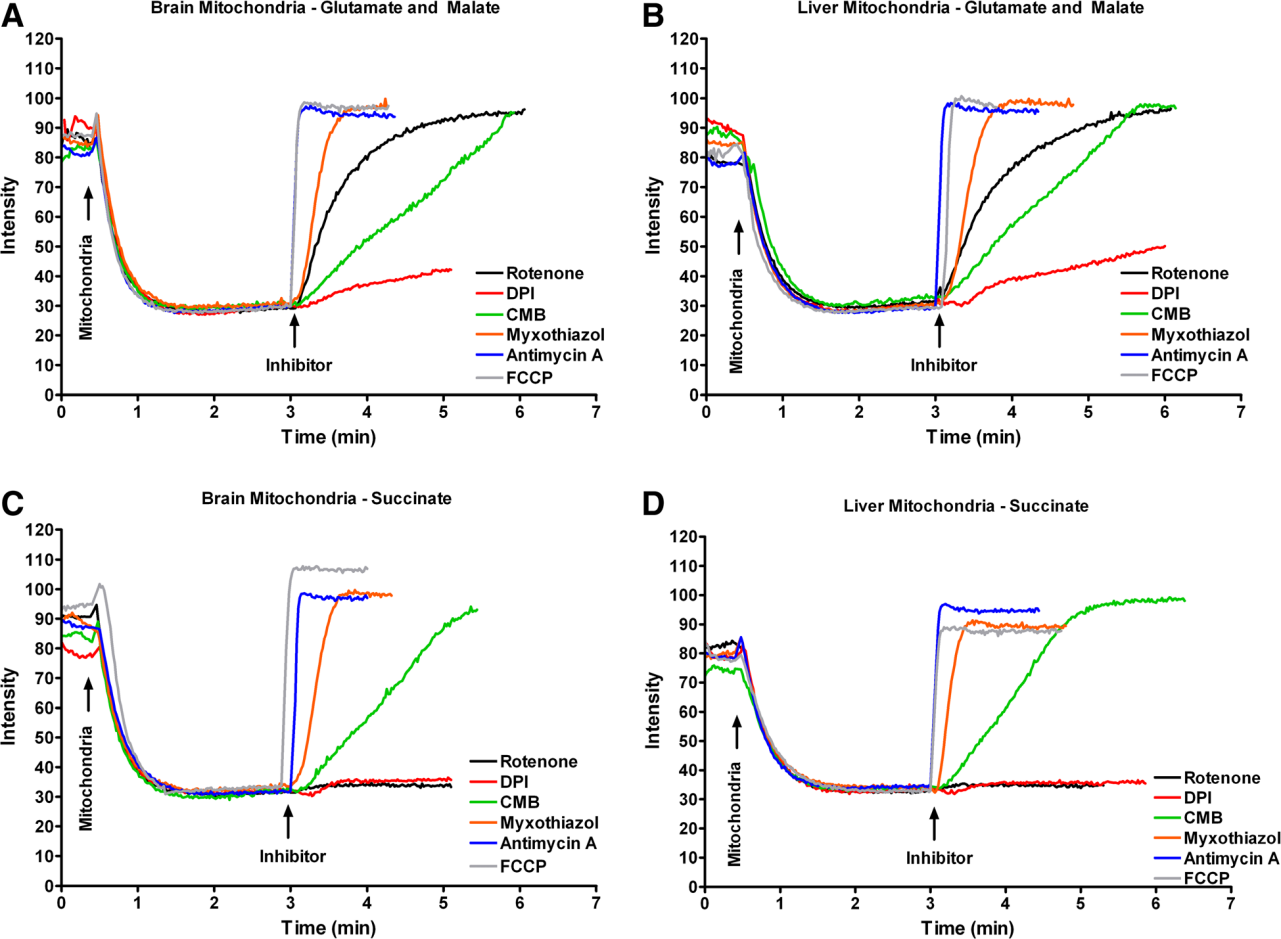
Mitochondrial membrane potential is not different comparing brain and liver mitochondria and is equally affected by ETC. inhibitors in both mitochondrial populations. Mitochondrial membrane potential was qualitatively assessed for brain and liver mitochondria in respiratory state 2 utilizing glutamate and malate (**a** and **b**, respectively) or succinate (**c** and **d**, respectively). Mitochondria (150 μg) were added where indicated and membrane potential was recorded. At three minutes, 10 μM rotenone (black), 10 μM DPI (red), 10 μM CMB (green), 10 μM myxothiazol (orange), 10 μg/mL antimcyin A (blue), or 0.4 μM FCCP (gray) were added. Traces are representative of three replicates

Mitochondrial inner membrane potential, Δψ_m_, was assessed qualitatively in liver and brain mitochondria utilizing complex I or complex II substrates while in respiratory state 2. There were no significant differences in basal Δψ_m_ between brain and liver mitochondria. With either complex I or complex II substrates both brain and liver mitochondria were completely and rapidly depolarized by the addition of the uncoupler FCCP, and by antimycin A. Myxothiazol also completely depolarized mitochondria in each experimental condition yet with slightly slower kinetics. Rotenone depolarized brain and liver mitochondria utilizing glutamate and malate yet with slightly slower kinetics than myxothiazol (Fig. 6a, b). As expected, rotenone did not depolarize brain or liver mitochondria utilizing succinate (Fig. 6c, d). CMB completely depolarized brain and liver mitochondria utilizing glutamate and malate or succinate, yet relatively slowly. DPI resulted in only a slight depolarization of liver and brain mitochondria utilizing glutamate and malate (Fig. 6a, b) and negligible depolarization utilizing succinate (Fig. 6c, d).

### NADH transport activity is increased in brain mitochondria

**Fig. 7:**
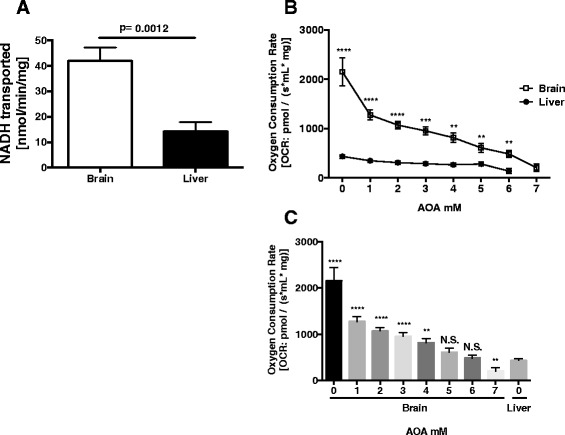
NADH-linked substrate transport is increased in brain compared with liver mitochondria. **a** The activity of the malate-aspartate shuttle was assessed by following the decrease in absorbance of NADH (0.14 mM) at 340 nM. After addition of mitochondria, baseline decrease in absorbance was recorded. Subsequently, further decrease of absorbance, indicative of malate-aspartate shuttle activity, was recorded after the addition of malate and glutamate. The data shown represent the average of three independent experiments performed in at least duplicate. **b** State 3 respiration supported by the complex I substrates glutamate and malate in the presence of ADP, with the stepwise (1 mM increments) addition of the malate aspartate shuttle inhibitor AOA. Traces were compiled from of three independent experiments performed in duplicate. **c** Brain mitochondrial state 3 respiration was determined in the presence of increasing concentrations of AOA in comparison with liver mitochondria in state 3 respiration without inhibition by AOA. Bars are representative of three independent experiments performed in duplicate. In 7b liver and brain mitochondria were compared for an individual dose of AOA. In 7c brain mitochondria at each AOA dose was compared to untreated liver mitochondria. Notations, ****-*p* < 0.0001, ***-*p* < 0.001, **-*p* < 0.01, N.S-Not significant

Increased respiration in brain mitochondria linked to complex I or complex II substrates cannot be attributed to enzymatic differences as the individual complex I, II, and III enzymatic activities showed no significant differences. However, differences in substrate transport could account for this difference. To determine if differences in transport of NADH into mitochondria of brain versus liver the autofluorescence of exogenously added NADH was followed. The rate of disappearance of NADH autofluorescence was calculated after the addition of ADP. This rate was corrected for the non-ADP linked rate, which indicated non-ETC. NADH use. NADH transport was increased approximately 3 fold in brain mitochondria compared with liver mitochondria (*p* = 0.0012) (Fig. 7a).

### Inhibition of the Malate-Aspartate Shuttle Differentially Regulates Respiration of Liver and Brain Mitochondria

Brain mitochondria have elevated rates of respiration as well as more rapid transport of NADH when compared to isolated liver mitochondria. To determine how the heightened respiration is controlled by substrate transport we utilized AOA, which inhibits the malate-aspartate shuttle though selective, competitive inhibition of aspartate amino transferases. For these studies we combined either brain or liver mitochondria with glutamate and malate. ADP was then added in excess to induce sustained maximal respiration (state 3). As expected, maximal state 3 respiration of brain mitochondria prior to addition of AOA was 2149 ± 309 [pmol O_2_/(s*mL*mg mt protein)] compared to a rate of 432.5 ± 38 in liver mitochondria (Fig. 7b). This elevation in respiration mirrors the difference in substrate transport (Fig. 7a). The final concentrations of AOA to achieve full inhibition of state 3 respiration were 7 mM for brain mitochondrial and 6 mM for liver mitochondria. As expected, the respiration rate of brain mitochondria was inhibited with increasing concentrations of AOA. Rates dropped by 41 % at 1 mM, 50 % (2 mM), 56 % (3 mM), 62 % (4 mM), 72 % (5 mM), 77 % (6 mM), and 91 % (7 mM) with all rates being statistically different compared to uninhibited state 3 respiration. As previously published [44], liver mitochondria were refractory to respiratory inhibition at lower concentrations of AOA. After addition of 1 mM AOA the O_2_ consumption by liver mitochondria fell by 20 % but was not significantly reduced versus liver mitochondria respiring in the absence of AOA. However, at doses of 2 mM and above respiration of liver was significantly inhibited dropping by 29 % (2 mM), and then 34 % (3 mM), 39 % (4 mM), 36 % (5 mM), and 69 % (6 mM). When comparing the AOA induced changes in respiration rates of brain to liver, the rate of brain mitochondrial O_2_ consumption was significantly different at all concentrations (Fig. 7b). Further, when comparing the inhibition of brain mitochondria to uninhibited liver mitochondria the rate of respiration of mitochondria isolated from brain was more rapid at doses of AOA between 1 mM and 4 mM (Fig. 7c). At a dose of 5 mM AOA the state 3 respiratory rate of brain mitochondria (607 ± 102) was no longer statistically distinguishable from AOA-free liver mitochondria. These data provide evidence that the higher substrate transport by brain mitochondria drives enhanced respiration and that through inhibition of the malate-aspartate transporter the rates of respiration of brain and liver mitochondria can be equalized.

### Gene expression of *Slc25a10* and Complex II–III coupled enzymatic activity

**Fig. 8:**
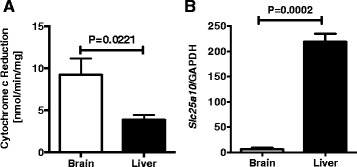
Complex II–III coupled enzymatic activity is increased in brain compared with liver mitochondria. **a** Complex II–III coupled enzymatic activity was assessed by following the reduction of ferricytochrome *c* to ferrocytochrome *c* at 550 nM by 40 μg of isolated mitochondria. A baseline was recorded after the addition of ferricytochrome *c* (15 μM), alamethacin (30 μg/mL), and rotenone (2 μM). The reaction was initiated by the addition of freshly isolated mitochondria (200 μg/mL) preincubated with succinate (20 mM) at 30 °C for 20 min in potassium phosphate buffer. Malonate (20 mM) was added in parallel wells, and the specific activity of complex II was obtained by subtracting the malonate insensitive activity. For brain mitochondria *n* = 9 and for liver mitochondria *n* = 8. **b** The expression level of *Slc25a10* was determined in brain and liver tissue, respectively, by RT-PCR and normalized to *Gapdh.* Three independent experiments were performed in triplicate

Substrate transport was further explored as the etiology for increased complex II-linked respiration in brain compared to liver mitochondria. *Slc25a10* encodes a dicarboxylate carrier protein found in the inner mitochondrial membrane that transports succinate or malate into the mitochondrial matrix in exchange for HPO_4_^2−^ [45]. *Slc25a10* expression was assessed by qRT-PCR. qRT-PCR revealed a significant increase in *Slc25a10* expression in liver compared with brain tissue (Fig. 8b). Given the lower *Slc25a10* expression in brain tissue, substrate transport via the dicarboxylate carrier cannot account for the increased respiratory activity of brain mitochondria when utilizing complex II substrates. While complex II and III activities were not significantly different between brain and liver mitochondria, the shuttling of electrons from complex II to complex III is not assessed by each individual assay and may underlie the differences in respiratory rates. By assessing the malonate sensitive reduction of cytochrome *c* after exogenous addition of succinate, brain mitochondria exhibited an approximately 2.5 fold higher complex II–III coupled enzymatic activity (*p* =0.0221) (Fig. 8a).

## Discussion

Mutations in the mitochondrial genome have been implicated as contributors to human disease and have been linked with diseases animal models. These illnesses can be the result of a homoplastic mutation in the mitochondrial population or as the result of heteroplasmy [46]. In either case patients do not exhibit a global failure of all tissues and are generally afflicted with tissue specific pathologies. Rossignol et al. have shown by determining metabolic control coefficients, that respiratory flux is regulated at different steps when comparing tissues. Due to these results they posited that these tissue specific regulatory distinctions account for the tissue sensitivity or specificity of a particular mitochondrial defect [47]. However, they found that rat liver and brain mitochondria are both primarily under the control of ATP synthase and the phosphate carrier [47], thus not providing an explanation for phenotypic changes observed herein between brain and liver mitochondria. We hypothesized that, in addition to metabolic control, the restriction of the pathology to specific tissues is likely affected by tissue specific mitochondrial respiratory function and ROS production as well as interactions between sequence variation in the mtDNA and alleles of nuclear encoded genes. Previously, we have demonstrated that by varying both the nuclear genome and the mtDNA, changes can be measured in mitochondrial ROS production [9, 10]. As an example, a transversion in *mt-Nd2* results in decreased mitochondrial ROS production in mice with tissue specific phenotypes and also interacts with loci within the nuclear genome [9, 10].

Determining the sites of ROS production within the mitochondrial ETC. requires the use of specific respiratory complex inhibitors that have distinct effects on the production of ROS. Interestingly, many widely used ETC. inhibitors have been shown in independent studies to have very distinct effects on the physiology of mitochondria isolated from different tissues. Tahara et al. studied the effects of a variety of ETC. inhibitors on ROS production in brain, heart, kidney, liver, and muscle tissue from rats [48]. However, the effects of ETC. inhibitors on mitochondrial function has not been well characterized in mouse models. In this paper we set out to characterize the effects of several ETC. inhibitors on mitochondria isolated from the commonly utilized C57BL/6 J mouse strain. We describe the effects of DPI, CMB, rotenone, myxothiazol, and antimycin A on liver and brain mitochondria by assaying ROS production, complex I, II, and III activities as well as mitochondrial oxygen consumption and membrane potential. Our results identify significant differences between liver and brain mitochondria, which are distinct from function of rat mitochondria. Furthermore, we propose a mechanistic explanation for increased respiratory rates in brain mitochondria.

Brain and liver mitochondria exhibited significant differences in mitochondrial ROS production (Fig. 2). Similar to rat brain mitochondria, mouse brain mitochondrial ROS production is stimulated by complex I and III inhibitors while utilizing complex I substrates. This increased ROS production is largely inhibited by the DPI corroborating the FMNs as a key site of ROS production [49–51]. The inhibition of ROS production by rotenone in mitochondria energized with succinate suggests that reverse electron flow to complex I redox centers is a major source of ROS production arising from reducing equivalents entering the ETC. through complex II in polarized mitochondria. In striking contrast, ROS production of liver mitochondria utilizing complex I or II substrates were either unaffected or slightly suppressed by these inhibitors.

The difference in effect of the inhibitors on liver mitochondria might be suspected to be due to differential inhibition of enzymatic activity. However, when assessing complex I (Fig. 3) or complex III (Fig. 4) enzymatic activity, each inhibitor had similar effects on the two tissues. Therefore, the underlying mechanisms of ROS production in brain and liver are likely distinct and may reflect the different energy requirement of each tissue. Mitochondria in highly energetically demanding neurons have evolved mechanisms by which basal ROS production is low, however ETC. inhibitors result in a drastic increase in ROS production which may be important in many neurodegenerative conditions, for instance the ability of rotenone to induce parkinsonism [52]. The finding that inhibition within the ETC in liver mitochondria did not cause significant increases in ROS production suggests the possibility that their redox centers are more sheltered from interaction with molecular oxygen. This may well represent an evolutionary adaptation by liver mitochondria given the relative abundance of toxins to which the liver can be exposed that could potentially impair mitochondrial function.

The effect of ETC. inhibitors on ROS production in brain mitochondria oxidizing succinate can largely be explained by their impact on Δψm. It is well known that ROS production is highly dependent upon Δψm, especially by brain mitochondria oxidizing succinate [43, 53]. We observed similar effects of the inhibitors on brain and liver mitochondria when respiration was supported by either complex I or II substrates (Fig. 6). Loss of Δψm likely impairs reverse electron flow to complex I, thereby reducing ROS production. That similar effects were not seen in liver mitochondria while the same degree of depolarization was achieved suggests that reverse electron flow does not play a major role in ROS production in liver mitochondria using complex II substrates. Importantly, the inhibition of ROS production in brain mitochondria oxidizing succinate reflects inhibition of FMN redox sites within complex I, as DPI was not shown to have an effect on Δψm with succinate as a substrate. This is in contrast to inhibition with rotenone, which binds distally within complex I and inhibits the reverse flow of electrons into complex I [54].

A caveat to the findings reported here is that brain ROS production results were derived from both neurons and glial cells, which could confound the interpretation of the ROS signal. Indeed, neurons and glial cells differ metabolically and may also differ in response to ETC. inhibitors. While the higher rates of oxidative metabolism in neurons might lead to the assumption that they will produce more ROS than glial cells, few studies have directly compared the rates of basal ROS production. Café et al. found no significant differences in basal ROS production between glial and neuronal cells although neurons were able to produce more ROS under conditions of heightened oxidative stress [55]. While it is possible to study ROS production in primary neuronal cultures as well as in glial cultures separately, increasing evidence suggests that the neuron-glial interaction is vital for the neuronal function [56, 57]. It has been shown that astrocytes provide metabolic support to neurons as well as providing structural support, modulating synaptic activity and neurotransmitter homeostasis, and detoxifying free radicals [58]. Furthermore, there is substantial variability among different types of neurons with regard to function, metabolism, and susceptibility to oxidative stress [59]. Most importantly, utilizing glutamate and malate as substrates, the vast majority of ROS production should derive from neurons. Indeed, the activity of the malate-aspartate shuttle has been demonstrated to be low if present at all in astrocytes [60] with the aspartate-glutamate carrier likely not present to a significant degree on glial cells [61].

Complex I inhibitors showed different degrees of inhibition of complex I supported respiration. Rotenone inhibited respiration to a greater extent than DPI and CMB (Fig. 5b, c), which is supported by our data showing greater inhibition of complex I enzyme activity by rotenone (Fig. 3b). DPI inhibited respiration in the presence of either complexes I or II substrates (Fig. 5), which is consistent with the ability of DPI to react with flavohydroquinones in both complexes I and II producing a hydroxyl radical leading to subsequent flavoprotein inactivation [62]. Indeed, we observed a similar reduction in succinate supported respiration by DPI as has been previously reported [63]. However, liver mitochondrial complex II supported respiration showed resistance to inhibition by DPI (Fig. 5d, e), which may be due to decreased inhibition of complex II but merits further investigation. The putative complex I inhibitor CMB also strongly inhibited succinate supported respiration in both brain and liver mitochondria (Fig. 5d, e). This results from the nearly complete ablation of complex III activity by CMB (4). CMB likely inhibits more broadly than merely complex I redox centers, potentially also having an inhibitory effect on the electron carrying capacity of complex III. Significant differences were also observed between brain and liver mitochondria with regard to the percent inhibition of state 3 respiration supported by complex I substrates in response to DPI and CMB (Fig. 5c), and state 3 respiration supported by complex II substrates in response to DPI and rotenone (Fig. 5e). This differential response to the inhibitors is not reflected in complex I enzymatic activity in response to CMP, DPI, and rotenone, or the inhibitors’ effects on mitochondrial membrane potential. Future studies to describe the mechanism of these compounds to differentially affect mitochondrial respiration may focus on their effects on supercomplex structure as well as on substrate availability.

State 2 and 3 respiratory activity comparing brain and liver mitochondria in the absence of inhibitors exhibited significant differences. Assaying either complex I or complex II supported respiration, brain mitochondrial oxygen consumption was approximately twice as high as liver mitochondrial oxygen consumption. It has been suggested that this may be secondary to increased complex I and complex II enzyme activities in mitochondria from brain relative to liver [64]. However, under our assay conditions, there were no significant differences between liver and brain mitochondria comparing the activities of complexes I, II, or III (Figs. 3, and 4a, b). The observation that both state 3 as well as state 2 respiration are twice as high in brain compared to liver mitochondria suggests that the difference is not due to variations in complex V (ATP synthase) activity. Additionally, addition of the complex V inhibitor oligomycin during state 3 respiration resulted in oxygen consumption rates twice as high in brain as in liver mitochondria (data not shown), further supporting the notion that upstream enzymatic activities were not responsible for the difference in respiratory rate. While the results herein assess membrane potential qualitatively and may not be able to detect minor changes, the ostensibly similar state 2 membrane potential between brain and liver mitochondria utilizing glutamate and malate or succinate make changes in membrane potential an unlikely cause of the disparate respiratory rates between the two tissues (Fig. 6).

Given the absence of differences in enzymatic activities or membrane potential, differences in substrate transport could account for differences in respiratory rates. Variation in the activity of the malate-aspartate shuttle between different tissues has been previously reported [65]. Indeed, a three-fold increase in NADH transport was observed in brain compared to liver mitochondria (Fig. 7). To support the notion that increased complex I respiration in brain mitochondria was due to increased substrate transport we inhibited the malate-aspartate shuttle with AOA. We showed that rates of respiration could be normalized with AOA treatment and also showed a greater degree of inhibition of brain mitochondrial respiration by AOA. The increased shuttle activity in brain mitochondria may be required to provide aspartate for N-acetylaspartate and myelin production [42]. Consistently, a homozygous mutation in the gene encoding the glutamate-aspartate carrier (*Slc25a12)* has been linked to psychomotor retardation, hypotonia, and hypomyelination of the central nervous system [66]. *Slc25a12* knockout mice have also been shown to have growth retardation, generalized tremor, motor deficits, decreased survival, and impaired myelination [42]. Importantly, variations in malate aspartate shuttle activity may differ in response to mitochondrial calcium fluxes [44], with transient calcium spikes during neuronal activity modulating shuttle activity. Therefore, further in vivo studies will be necessary to further characterize the interplay between substrate delivery and respiration under different physiological conditions.

Differences in complex II supported respiration cannot be attributed to differences in substrate transport. As noted above, the gene expression of *Slc25a10* was lower in brain compared to liver mitochondria. There was also no observed difference in complex II enzymatic activity between brain and liver mitochondria (Fig. 4a). However, there was a 2.5 fold increase in complex II–III coupled enzymatic activity (Fig. 8a), providing an explanation for increased complex II driven respiration in brain mitochondria. This is consistent with a previous report indicating increased complex II–III coupled enzymatic activity in rat brain compared with liver mitochondria [36]. However, the mechanism underlying differences in complex II–III coupled enzymatic activity remains unclear. Complex II is known to physically associate with complex III [67], and the association of respiratory complexes has been shown to regulate electron flux and respiratory rates [68]. This interaction may differ between brain and liver mitochondria, accounting for the difference in coupled enzymatic activity. Furthermore, in comparison with liver mitochondria, brain mitochondria do not maintain a storage pool of intermediates including ubiquinone [38], which could drastically alter the coupled complex II–III activity, the rate limiting step which relies upon the reduction of ubiquinone.

Increased complex II supported respiration in brain is consistent with previous reports; however, complex I supported respiration has been shown to be slightly higher in rat liver mitochondria compared with rat brain mitochondria [48]. Thus, the increased complex I supported respiration in brain and the mechanism of increased NADH transport distinguishes mouse from rat mitochondria. Whether this mechanism exists in other species merits further investigation.

## Conclusions

In this paper we have demonstrated important differences between mouse brain and liver mitochondria regarding their responses to ETC. inhibitors. Supported by complex I substrates, brain mitochondria produce elevated levels of ROS when either complex I or III is inhibited, while liver mitochondrial ROS production is unchanged in the presence of the same inhibitors. Additionally, the drastically elevated ROS levels in mouse brain mitochondria utilizing succinate as a substrate is inhibited to a greater extent by DPI and CMB than by rotenone, myxothiazol, or antimycin A. This elevated ROS is not present at all when assaying liver mitochondria utilizing succinate and inhibitors result in a slight or no reduction in ROS production. Many of these effects can be explained in the context of the impact of these inhibitors on complex I and III enzymatic activity, respiration, and membrane potential. We further demonstrated that increased respiration in brain mitochondria can in part be explained by increased substrate transport to support complex I activity, and increased coupled complex II–III activity while utilizing complex II substrates. These results should be considered when assaying mouse mitochondria in the presence of electron transport chain inhibitors.

## Methods

### Animals

C57BL/6 J (B6) mice were purchased from The Jackson Laboratory (Bar Harbor, ME). Mice were maintained in the Animal Care Services research animal facility at the University of Florida. All mice were bred and maintained in a specific pathogen-free vivarium and allowed free access to food (Teklad Irradiated LM-485 Mouse/Rat Diet, Harlan Teklad, Madison, WI) and drinking water. All procedures involving animals were approved by the University of Florida Institutional Animal Care and Use Committee and were in compliance with “Principles of Laboratory Animal Care” and the current laws of the United States of America.

### Isolation of mitochondria from brain and liver

Brain mitochondria were isolated previously as described [9, 69]. Briefly, Percoll dilutions were prepared in isolation buffer I (IB I) (225 mM Mannitol, 75 mM Sucrose, 10 mM HEPES Potasium, 1 mM EDTA, 0.1 % fatty acid free BSA, pH 7.4). All steps were performed at 4 °C. The whole brain was excised, and homogenized in 12 % Percoll, layered on top of a gradient of 24 % and 42 % Percoll, and centrifuged at 27,000 × *g* for 10 min. The mitochondrial fraction, located between the 24 % and 42 % layers, was removed with a syringe, diluted in IB I, and centrifuged at 10,000 × *g* for 10 min. The supernatant was discarded, and the pellet was re-suspended in isolation buffer II (IB II) (225 mM Mannitol, 75 mM Sucrose, 10 mM HEPES Potasium, 0.1 mM EDTA, pH 7.4) and centrifuged at 10,000 × *g* for 10 min. The supernatant was again discarded, and the pellet was re-suspended in a total volume of 100 μL IB II. Protein concentrations for brain mitochondria as well as liver mitochondria preparations (described below) were determined using the BCA protein assay (Pierce, Rockford, IL).

Liver mitochondria were isolated by differential centrifugation previously as described [10] and also using a Percoll gradient. All steps were performed at 4 °C. For isolation by differential centrifugation livers were removed, washed in IB II, and then homogenized in IB I. The homogenate was centrifuged at 1300 × *g* for 10 min. The supernatant was saved and centrifuged at 10,000 × *g* for 10 min. The supernatant was discarded and the pellet was re-suspended in IB II and spun at 10,000 × *g* for 10 min. The resulting pellet was re-suspended in approximately 100 μL IB II. For isolation of liver mitochondria using Percoll, livers were removed, washed in IB II, and then homogenized in IB I. The homogenate was centrifuged at 1300 × *g* for 10 min, the supernatant saved, and centrifuged at 10,000 × *g* for 10 min. Resulting pellets were resuspended in 15 mL of 12 % Percoll, layered on top of a gradient of 24  and 42 % Percoll, and centrifuged at 27,000 × *g* for 10 min. The mitochondrial fraction located between the 24  and 42 % layers was removed with a syringe, diluted in IB I, and centrifuged at 10,000 × *g* for 10 min. The supernatant was discarded, and the pellet was re-suspended in IB II and centrifuged at 10,000 × *g* for 10 min. The resulting pellet was re-suspended in 100 μL IB II.

Mitochondrial purity was determined by subjecting isolated mitochondrial samples to Western blot analysis. Mitochondrial preparations were suspended in 100 μL lysis buffer (50 mM Tris base, 137 mM NaCl, 10 % glycerol, 1 % NP-40, 1 M NaF, 5 mg/ml leupeptin, 5 mg/ml aprotinin, 0.1 M Na_3_PO_4_, 0.33 M Phenylmethanesulfonyl fluoride). Each protein sample (25 μg) was combined with an equal volume of Laemmli Sample Buffer (Bio-Rad, Hercules, CA), and boiled at 100 °C for 5 min. Samples were separated on 12 % SDS-PAGE gels and transferred to nitrocellulose, using standard procedures [70]. Western blotting was performed with either an anti-cytochrome c oxidase subunit IV (COX IV) antibody (Abcam, Cambridge, MA) or an anti-glyceraldehyde-3-phosphate dehydrogenase (GAPDH) antibody (Sigma, St. Louis, MO) followed by horseradish peroxidase-conjugated Ig and detection with enhanced chemiluminescence (Amersham International, Amersham, UK). Chemiluminescence was detected with a FluorChem HD2 (Alpha Innotech, Santa Clara, CA) and the images were collected and analyzed using AlphaEaseFC software (Alpha Innotech).

### Measurements of mitochondria reactive oxygen species production and mitochondrial transmembrane potential

Mitochondrial ROS production was assessed as previously described [9, 10]. All assays were performed with constant stirring at 37 °C using 150 μg of mitochondrial protein in 500 μL incubation media (IM) (125 mM KCL, 2 mM K_2_HPO_4_, 5 mM MgCl_2_, 10 mM HEPES, 10 μM EGTA, pH 7.2) in respiratory state 2 supplemented with either 5 mM L-glutamate and 5 mM L-malate or 5 mM succinate. ROS production was measured by continuously recording the real-time oxidation of 2 μM Amplex Red (Molecular Probes, Eugene, OR) to the fluorescent molecule Resorufin, catalyzed by 1 U/mL horseradish peroxidase. The use of Amplex Red primarily detects ROS in the form of hydrogen peroxide [71]. Endogenous dismutase activity was likely sufficient to metabolize all of the superoxide produced in this system to hydrogen peroxide as the addition of exogenous superoxide dismutase did not increase the signal [43]. Fluorescence was measured with an excitation wavelength of 560 nm (slit 1.5 nm) and an emission wavelength of 590 nm (slit 3 nm) using an RF-5301PC spectrofluorophotometer (Shimadzu, Kyoto, Japan). Inhibitors of the electron transport chain were added at the indicated concentrations. To determine the optimal concentration of each inhibitor, titration curves using 0.1, 1, 5, 10, and 20 μM final concentrations of each inhibitor were performed. ROS production was equal for the 5, 10, and 20 μM doses of Complex I and Complex III inhibitors in both brain and liver mitochondria (Additional file 1: Table S1). For the studies included here we chose 10 μM of each inhibitor (Fig. 2). Rates of ROS production were measured for at least 2 min for each treatment. All rates were linear over the time interval measured. Vehicle alone (DMSO at any concentration used) did not significantly alter ROS production from either liver or brain mitochondria utilizing glutamate and malate or succinate (Additional file 1: Table S2). Slopes were converted into units of H_2_O_2_ (pmol / min / mg) as previously described [71]. As vehicle did not alter the rate of basal ROS production, ROS production after addition of ETC. inhibitor was compared to the rate in incubation media alone.

Mitochondrial transmembrane potential (Δψ_m_) was measured using the fluorescence quenching of cationic dye safranin O (2.5 μM). Mitochondria (150 μg) were added to incubation media supplemented with either 5 mM L-glutamate and 5 mM L-malate or 5 mM succinate followed by the addition of 2.5 μM safranin O. Fluorescence was measured at excitation wavelength 495 nm (slit 3 nm) and emission wavelength 586 nm (slit 10 nm).

### Complex I (NADH:ubiquinone oxidoreductase) enzymatic activity

Complex I activity was measured by monitoring the oxidation of NADH at 340 nm as well as the reduction of ubiquinone at 280 nm using a Spectramax E5 spectrofluorophotometer (Molecular Devices). The assay medium contained potassium phosphate (25 mM, pH 7.2 at 20 °C), 5 mM MgCl_2_, 2.5 mg/mL bovine serum albumin (fraction V), and 2 mM KCN. Each reaction was supplemented with 0.13 mM NADH, 65 μM ubiquinone_1_, and 10 μg/mL antimycin A in a total volume of 200 μL. The reaction was initiated by the addition of freeze fractured mitochondria (40 μg), and the rates of oxidation of NADH and reduction of ubiquinone were simultaneously recorded. In parallel wells, either 10 μM rotenone, 10 μM DPI, or 10 μM CMB was added. The rate of change in absorbance was measured for 3 minutes [72].

### Complex II (succinate:ubiquinone oxidoreductace) enzymatic activity

Complex II activity was measured by monitoring the reduction of 2,6-dichloroindophenolate (DCIP) at 600 nm using a Spectramax E5 spectrofluorophotometer (Molecular Devices). The assay medium contained potassium phosphate (25 mM, pH 7.2 at 20 °C), 5 mM MgCl_2_, and 20 mM sodium succinate. In parallel wells, malonate (20 mM) was added. Mitochondria (8 μg/mL) were incubated in the assay medium at 30 °C for 10 min. A baseline was recorded for 1 min after the addition of 2 μg/ml antimycin A, 2 μg/ml rotenone, 2 mM KCN, and 50 M 2,6-dichloroindophenolate in a total volume of 200 μL. The reaction was initiated by the addition of 65 μM ubiquinone_1_, and the rate of reduction of 2,6-dichloroindophenolate was recorded for 3 min. The complex II specific rate was determined by subtracting the malonate insensitive activity from the total activity [72].

### Complex II-Complex III coupled enzymatic activity

Complex II-III activity was determined by monitoring the reduction of ferricytochrome *c* to ferrocytochrome *c* at 550 nm using a Spectramax E5 spectrofluorophotometer (Molecular Devices). Alamethicin was used to permit cytochrome *c* binding within complex III while maintain the integrity of the ETC. [73, 74]. The assay medium contained potassium phosphate (25 mM, pH 7.2 at 20 °C), 2.5 mM MgCl_2_, 2.5 mg/mL bovine serum albumin, and 2 mM KCN. A baseline was recorded for 1 min at 550 nm after the addition of alamethicin (30 μg/mL), ferricytochrome c (15 μM), and rotenone (2 μM) in a total volume of 200 μL. In parallel wells, malonate (20 mM) was added. Reactions were initiated with the addition of freshly isolated mitochondria preincubated with succinate (20 mM) at 30 °C for 20 min in potassium phosphate buffer. The rate of reduction was recorded for 3 min. The coupled complex II–III specific rate was determined by subtracting the malonate sensitive activity from the total activity [75].

### Complex III (cytochrome c reductase) enzymatic activity

Complex III activity was determined by monitoring the reduction of ferricytochrome *c* at 550 nm using a Spectramax E5 spectrofluorophotometer (Molecular Devices). The assay medium contained potassium phosphate (25 mM, pH 7.2 at 20 °C), 5 mM MgCl_2_, 2.5 mg/mL bovine serum albumin (fraction V), and 2 mM KCN. The inclusion of KCN precluded the re-oxidation of fererocytochrome *c* by cytochrome *c* oxidase. Each reaction was supplemented with 15 μM ferricytochrome *c*, 10 μM rotenone, 0.6 mM n-dodecyl-β-D-maltoside, and 35 μM ubiquinol_2_ in a total volume of 200 μL. Ubiquinol_2_ was prepared by dissolving 8 μg of ubiquinone in 1 mL of ethanol; the solution was adjusted to pH 2 with 6 M HCl. Ubiquinone was reduced using excess sodium borohydride. Ubiquinol_2_ was extracted into 2:1 (v/v) diethylether:cyclohexane, evaporated under nitrogen gas, dissolved in 1 mL ethanol, and acidified to pH 2 with 6 M HCl. Reactions were initiated by the addition of mitochondria (20 μg) and the rate of reduction of ferricytochrome *c* to ferrocytochrome *c* was recorded for one minute. The rate was calculated based on the first 10–15 s, which remained linear. In duplicate wells either 10 μM antimycin A, 10 μM DPI, or 10 μM CMB was added [72].

### Oxygen consumption

Oxygen consumption was assessed using an Oxygraph-2 k (Oroboros Instruments, Innsbruck, Austria). For each measurement the chamber volume was 2 mL and a total of 0.4 mg of mitochondrial protein was added to MiR05 respiration medium. State 2 respiration was recorded after the addition of mitochondria. State 3 respiration was induced by the addition of ADP (0.5 mM final concentration). After maximum activated respiration was reached, the indicated inhibitor was added. All rates were recorded for at least three minutes.

Mitochondrial respiration was also studied after the addition of aminooxyacetate (AOA), which inhibits the malate-aspartate shuttle by inhibiting aspartate amino transferases [76]. State 2 respiration was recorded after the addition of complex I substrates glutamate and malate. State 3 respiration was induced with ADP at 1 mM final concentration to generate a sustained activated respiratory state. AOA was introduced stepwise into the reaction chamber in additive 1 mM doses, 3 min apart, until respiration was inhibited to levels equal to or less than state 2 respiration.

### Measurement of malate-aspartate shuttle activity

The capacity to transport NADH by the malate-aspartate shuttle was assessed in both isolated brain and liver mitochondria by following the decrease in NADH fluorescence at 340 nm using an RF-5301PC spectrofluorophotometer (Shimadzu) as previously described [77].

### RT-PCR

RNA was purified from brain and liver using TRIzol Reagent (Life Technologies, Rockville, MD). cDNA was generated using the SUPERSCRIPT Choice System (Life Technologies, Carlsbad, CA). Primer sets, specific for *Slc25a10*, *mt-Cyb*, *mt-Co2*, and *Actb* (beta-Actin) [78] were purchased from Qiagen (Valencia, CA). Tests were run on a LightCycler 480 (Roche, Indianapolis, IN), using SYBR Green Supermix (BioRad, Hercules, CA) for detection as per manufacturer’s protocol in a total volume of 50 μL. All PCR consisted of an initial 10 min denaturation step at 95 °C, followed by 45 two-step cycles of (95 °C for 10 s, 60 °C for 30 s). Detection was performed during each 60 °C extension step. After the 45th cycle a melt-curve was run to assess specificity. To further determine specific amplification the reaction products were run on 4 % agarose gels as previously described [79, 80].

### Chemicals

All chemicals were obtained from Sigma-Aldrich Co. unless otherwise noted. Stock solutions of rotenone and antimycin A were prepared in ethanol. Other ETC. inhibitor stock solutions were prepared in dimethyl sulfoxide (DMSO). All working dilutions of ETC. inhibitors were prepared in incubation media.

### Data analysis

Statistical analysis was performed using Prism 5 (Graphpad Software, La Jolla, CA). Data are represented as means ± SEM, with means considered to be significantly different at *p* < 0.05.

## Additional file

Additional file 1: Table 1. Effect of Mitochondrial Complex I or Complex III Inhibitor Dose on Liver or Brain Mitochondrial ROS Production When Respiring on Complex I or Complex II Substrates. **Table 2.** Effect of DMSO Concentration on Liver or Brain Mitochondrial ROS Production When Respiring on Complex I or Complex II Substrates. (DOCX 20 kb)
