# Supplementary material for: Respiration and substrate transport rates as well as reactive oxygen species production distinguish mitochondria from brain and liver
Source: BMC Biochem. 2015 Sep 10;16:22. doi: 10.1186/s12858-015-0051-8 (PMC4564979; doi:10.1186/s12858-015-0051-8)
Supplement: Additional file 1: Table 1. — Effect of Mitochondrial Complex I or Complex III Inhibitor Dose on Liver or Brain Mitochondrial ROS Production When Respiring on Complex I or Complex II Substrates. Table 2. Effect of DMSO Concentration on Liver or Brain Mitochondrial ROS Production When Respiring on Complex I or Complex II Substrates. (DOCX 20 kb) [file 12858_2015_51_MOESM1_ESM.docx]

Table 1. Effect of Mitochondrial Complex I or Complex III Inhibitor Dose on Liver or Brain Mitochondrial ROS Production When Respiring on Complex I or Complex II Substrates

Concentration Complex I Substrates Complex II Substrate

of Rotenone (µM) Brain Liver Brain Liver

0 100 ± 15 ^a^ 194 ± 42 ^a^ 1192 ± 316 ^a^ 278 ± 52 ^a^

0.1 105 ± 9 ^a^ 172 ± 42 ^a,b^ 954 ± 219 ^a^ 282 ± 22 ^a^

1 512 ± 101 ^b^ 159 ± 43 ^a,b^ 477 ± 215 ^b^ 232 ± 63 ^a,b^

5 1199 ± 182 ^c^ 122 ± 13 ^b^ 421 ± 174 ^b^ 198 ± 32 ^a,b^

10 1173 ± 253 ^c^ 117 ± 14 ^b^ 432 ± 144 ^b^ 181 ± 40 ^a,b^

20 1213 ± 177 ^c^ 119 ± 23 ^b^ 309 ± 115 ^b^ 174 ± 40 ^a,b^

Concentration Complex I Substrates Complex II Substrate

of Myxothiazol (µM) Brain Liver Brain Liver

0 126 ± 28 ^a^ 213 ± 31 1192 ± 322 ^a^ 224 ± 34

0.1 128 ± 32 ^a^ 187 ± 25 1200 ± 343 ^a,b^ 205 ± 37

1 615 ± 198 ^b^ 196 ± 33 802 ± 235 ^a,b^ 190 ± 37

5 658 ± 73 ^b^ 227 ± 48 655 ± 344 ^b,c^ 201 ± 58

10 726 ± 142 ^b^ 200 ± 51 543 ± 156 ^c^ 196 ± 34

20 778 ± 187 ^b^ 201 ± 21 555 ± 107 ^c^ 235 ± 98

Concentration Complex I Substrates Complex II Substrate

of Antimycin A (µM) Brain Liver Brain Liver

0 99 ± 15 ^a^ 205 ± 80 1265 ± 277 ^a^ 283 ± 66

0.1 134 ± 28 ^a^ 211 ± 40 847 ± 188 ^a,b^ 287 ± 46

1 651 ± 139 ^b^ 204 ± 71 655 ± 322 ^b^ 277 ± 75

5 691 ± 179 ^b^ 187 ± 30 532 ± 212 ^b^ 240 ± 74

10 626 ± 105 ^b^ 145 ± 32 607 ± 133 ^b^ 239 ± 72

20 668 ± 88 ^b^ 118 ± 33 488 ± 188 ^b^ 157 ± 21

To determine the impact of electron transport complex I and III inhibitors on mitochondrial ROS production, a dose response titration curve was preformed. Mitochondrial ROS production was assessed utilizing isolated brain mitochondria respiring on the complex I substrates glutamate and malate, isolated liver mitochondria respiring on the complex I substrates glutamate and malate, isolated brain mitochondria respiring on the complex II substrate succinate, isolated liver mitochondria respiring on the complex II substrate succinate. Basal ROS production was recorded for three minutes. After this time, either a complex I inhibitor (rotenone) or a complex III inhibitor (myxothiazol or antimycin A) was added at the indicated concentration and ROS production was recorded for an additional two minutes. A total of 150 µg of isolated mitochondria was used for each experiment. The mean ± SD from a minimum of 3 runs is presented here for each data point. One-way ANOVA was used to compare the effect of dose of each inhibitor on the rate of ROS production by brain or liver mitochondrial respiring on complex I or complex II substrates. Within each column, different letters represent statistical significance at a level of P<0.05. If letters do not appear in a column there was no calculated statistical significance.

Table 2. Effect of DMSO Concentration on Liver or Brain Mitochondrial ROS Production When Respiring on Complex I or Complex II Substrates

Concentration Complex I Substrates Complex II Substrate

of DMSO (µM) Brain Liver Brain Liver

0 134 ± 29 176 ± 45 1151 ± 307 235 ± 113

0.0128 145 ± 45 182 ± 13 1187 ± 471 224 ± 123

0.128 148 ± 40 174 ± 11 1085 ± 181 209 ± 115

0.64 158 ± 57 172 ± 16 1115 ± 287 202 ± 107

1.28 134 ± 48 176 ± 29 1361 ± 335 214 ± 103

2.56 178 ± 58 160 ± 20 1163 ± 221 311 ± 153

12.8 159 ± 24 163 ± 38 1182 ± 95 158 ± 85

To determine the effect of DMSO on mitochondrial ROS production, a dose response titration curve was preformed. Mitochondrial ROS production was assessed utilizing isolated brain mitochondria respiring on the complex I substrates glutamate and malate, isolated liver mitochondria respiring on the complex I substrates glutamate and malate, isolated brain mitochondria respiring on the complex II substrate succinate, isolated liver mitochondria respiring on the complex II substrate succinate. Basal ROS production was recorded for three minutes. After this time, DMSO alone was added at the indicated concentration and ROS production was recorded for an additional two minutes. A total of 150 µg of isolated mitochondria was used for each experiment. A minimum of 3 runs were completed for each data point presented here. The mean ± SD from a minimum of 3 runs is presented here for each data point. One-way ANOVA was used to compare the effect DMSO dose on the rate of ROS production by brain or liver mitochondrial respiring on complex I or complex II substrates. Statistical significance was not calculated for any condition reported here.
